# Supplementary material for: Ovothiol A is the Main Antioxidant in Fish Lens
Source: Metabolites. 2019 May 10;9(5):95. doi: 10.3390/metabo9050095 (PMC6572425; doi:10.3390/metabo9050095)
Supplement: Supplementary file 1 [file metabolites-09-00095-s001.pdf]

Supplementary Material

for

Ovothiol A is the main antioxidant in the fish lens

Vadim V. Yanshole[1,2], Lyudmila V. Yanshole[1,2], Ekaterina A. Zelentsova[1,2], Yuri P. Tsentalovich[1,2*]

[1]International Tomography Center SB RAS, Institutskaya 3a, Novosibirsk 630090, Russia

[2]Novosibirsk State University, Pirogova 2, Novosibirsk 630090, Russia

*e-mail: yura@tomo.nsc.ru

Table of content

Supplementary Table S1. Characterization of fish lenses used in this study.

Supplementary Table S2. Concentrations of histidine, ovothiol and glutathione in individual lenses of *S. lucioperca* and *R. rutilus lacustris*.

Supplementary Figure S1. NMR spectrum of ovothiol A (OSH).

Supplementary Figure S2. NMR spectrum of oxidized ovothiol A (OSSO).

Supplementary Figure S3. ESI-Q-TOF mass-spectrum of CID fragments (MS/MS) of isolated parent ion with m/z 202.0643 (OSH).

Supplementary Figure S4. ESI-Q-TOF mass-spectrum of CID fragments (MS/MS) of isolated parent ion with m/z 401.1061 (OSSO).

Table S1. Characterization of fish lenses used in this study.

Sample Number	Fish Type	Date of catching	State of water area	Lens weight, mg
Group 1				
SA1	*S. lucioperca*	10.10.2017	Open water	158
SA2	*S. lucioperca*	10.10.2017	Open water	154
SA3	*S. lucioperca*	10.10.2017	Open water	94
SA4	*S. lucioperca*	10.10.2017	Open water	75
SA5	*S. lucioperca*	10.10.2017	Open water	135
SA6	*S. lucioperca*	10.10.2017	Open water	94
SA7	*S. lucioperca*	10.10.2017	Open water	79
SA8	*S. lucioperca*	10.10.2017	Open water	140
Group 2				
SW1	*S. lucioperca*	02.02.2018	Ice	98
SW2	*S. lucioperca*	02.02.2018	Ice	79
SW3	*S. lucioperca*	02.02.2018	Ice	78
SW4	*S. lucioperca*	02.02.2018	Ice	109
SW5	*S. lucioperca*	02.02.2018	Ice	114
SW6	*S. lucioperca*	02.02.2018	Ice	80
SW7	*S. lucioperca*	02.02.2018	Ice	73
Group 3				
RA1	*R. rutilus lacustris*	28.11.2017	Ice	47
RA2	*R. rutilus lacustris*	28.11.2017	Ice	33
RA3	*R. rutilus lacustris*	28.11.2017	Ice	49
RA4	*R. rutilus lacustris*	28.11.2017	Ice	42
RA5	*R. rutilus lacustris*	28.11.2017	Ice	35
RA6	*R. rutilus lacustris*	28.11.2017	Ice	32
RA7	*R. rutilus lacustris*	28.11.2017	Ice	49
RA8	*R. rutilus lacustris*	28.11.2017	Ice	35
RA9	*R. rutilus lacustris*	28.11.2017	Ice	38
RA10	*R. rutilus lacustris*	28.11.2017	Ice	43
Group 4				
RW1	*R. rutilus lacustris*	09.02.2018	Ice	35
RW2	*R. rutilus lacustris*	09.02.2018	Ice	35
RW3	*R. rutilus lacustris*	09.02.2018	Ice	42
RW4	*R. rutilus lacustris*	09.02.2018	Ice	34
RW5	*R. rutilus lacustris*	09.02.2018	Ice	34

Table S2. Concentrations of histidine, ovothiol and glutathione in individual lenses of *S. lucioperca* and *R. rutilus lacustris* (in nmoles per gram of the lens wet weight).

Sample Number	Fish Type	Date of catching	Histidine	OSH	GSH
Group 1					
SA1	*S. lucioperca*	10.10.2017	887	3036	467
SA2	*S. lucioperca*	10.10.2017	877	2723	309
SA3	*S. lucioperca*	10.10.2017	722	3273	358
SA4	*S. lucioperca*	10.10.2017	862	3160	366
SA5	*S. lucioperca*	10.10.2017	734	2674	539
SA6	*S. lucioperca*	10.10.2017	791	3306	774
SA7	*S. lucioperca*	10.10.2017	901	2958	371
SA8	*S. lucioperca*	10.10.2017	887	2764	548
Group 2					
SW1	*S. lucioperca*	02.02.2018	344	1774	564
SW2	*S. lucioperca*	02.02.2018	321	1544	454
SW3	*S. lucioperca*	02.02.2018	349	1654	579
SW4	*S. lucioperca*	02.02.2018	324	1507	403
SW5	*S. lucioperca*	02.02.2018	317	1638	369
SW6	*S. lucioperca*	02.02.2018	324	1532	481
SW7	*S. lucioperca*	02.02.2018	339	1758	554
Group 3					
RA1	*R. rutilus lacustris*	28.11.2017	370	1156	127
RA2	*R. rutilus lacustris*	28.11.2017	306	939	315
RA3	*R. rutilus lacustris*	28.11.2017	387	1217	66
RA4	*R. rutilus lacustris*	28.11.2017	208	911	91
RA5	*R. rutilus lacustris*	28.11.2017	468	1301	406
RA6	*R. rutilus lacustris*	28.11.2017	291	857	343
RA7	*R. rutilus lacustris*	28.11.2017	409	1180	365
RA8	*R. rutilus lacustris*	28.11.2017	444	1173	382
RA9	*R. rutilus lacustris*	28.11.2017	296	1081	409
RA10	*R. rutilus lacustris*	28.11.2017	266	927	329
Group 4					
RW1	*R. rutilus lacustris*	09.02.2018	277	390	195
RW2	*R. rutilus lacustris*	09.02.2018	241	162	252
RW3	*R. rutilus lacustris*	09.02.2018	272	271	215
RW4	*R. rutilus lacustris*	09.02.2018	243	252	45
RW5	*R. rutilus lacustris*	09.02.2018	271	256	48

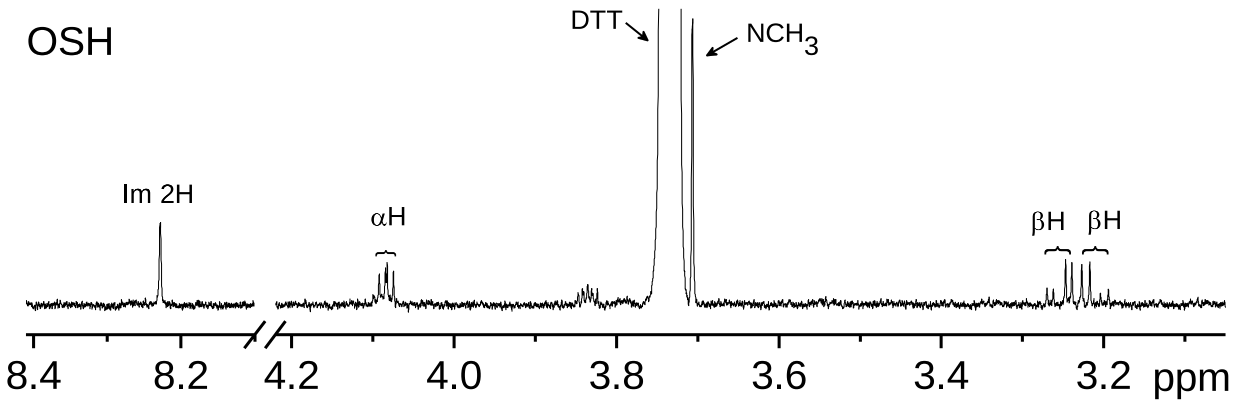

Figure S1. NMR spectrum of ovothiol A (OSH). ^{1}H NMR (700 MHz, D_2O): 3.210 (1H, dd, J = 6.9, 16.0 Hz, βH); 3.255 (1H, dd, J = 5.4, 16.0 Hz, βH); 3.707 (3H, d, J = 0.5 Hz, NCH_3); 4.083 (1H, dd, J = 5.4, 6.9 Hz, αH); 8.228 (1H, q, J = 0.5 Hz, Im 2H).

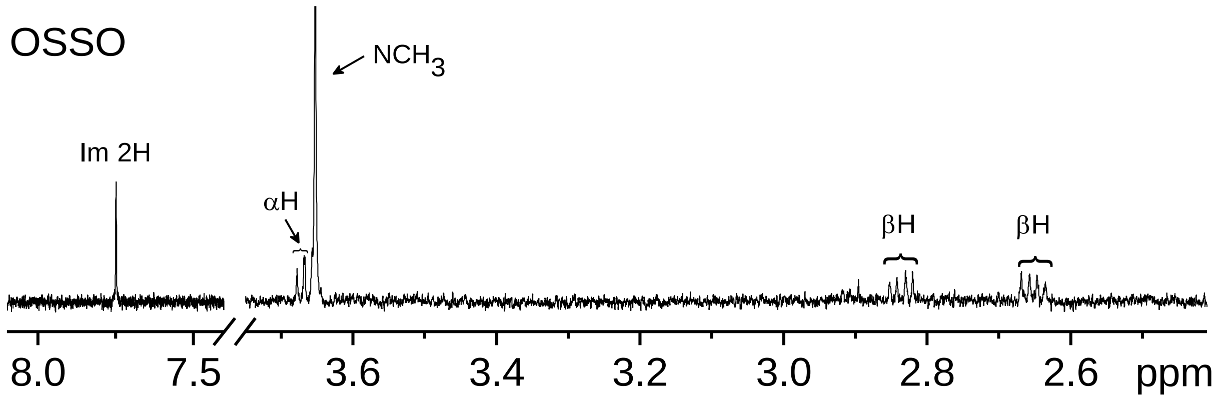

Figure S2. NMR spectrum of oxidized ovothiol A (OSSO). ^{1}H NMR (700 MHz, D_2O): 2.653 (1H, dd, J = 8.0, 15.6 Hz, βH); 2.836 (1H, dd, J = 6.9, 15.6 Hz, βH); 3.653 (3H, s, NCH_3); 3.668 (1H, dd, J = 6.9, 8.0 Hz, αH); 7.750 (1H, s, Im 2H).

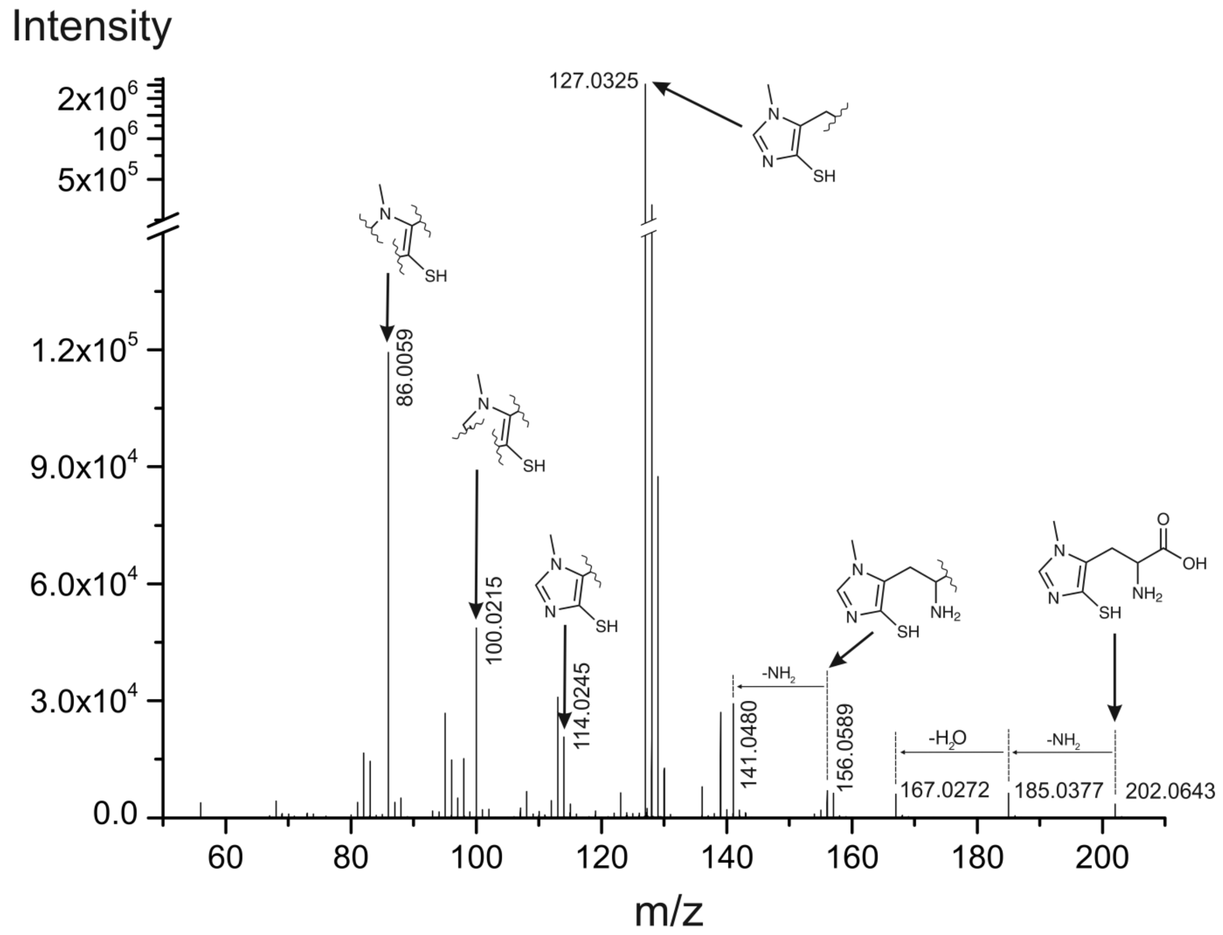

Figure S3. ESI-Q-TOF mass-spectrum of CID fragments (MS/MS) of isolated parent ion with m/z 202.0643 (OSH). Collision energy 35eV, positive ion mode. Wavy lines indicate the positions of the bond cleavage.

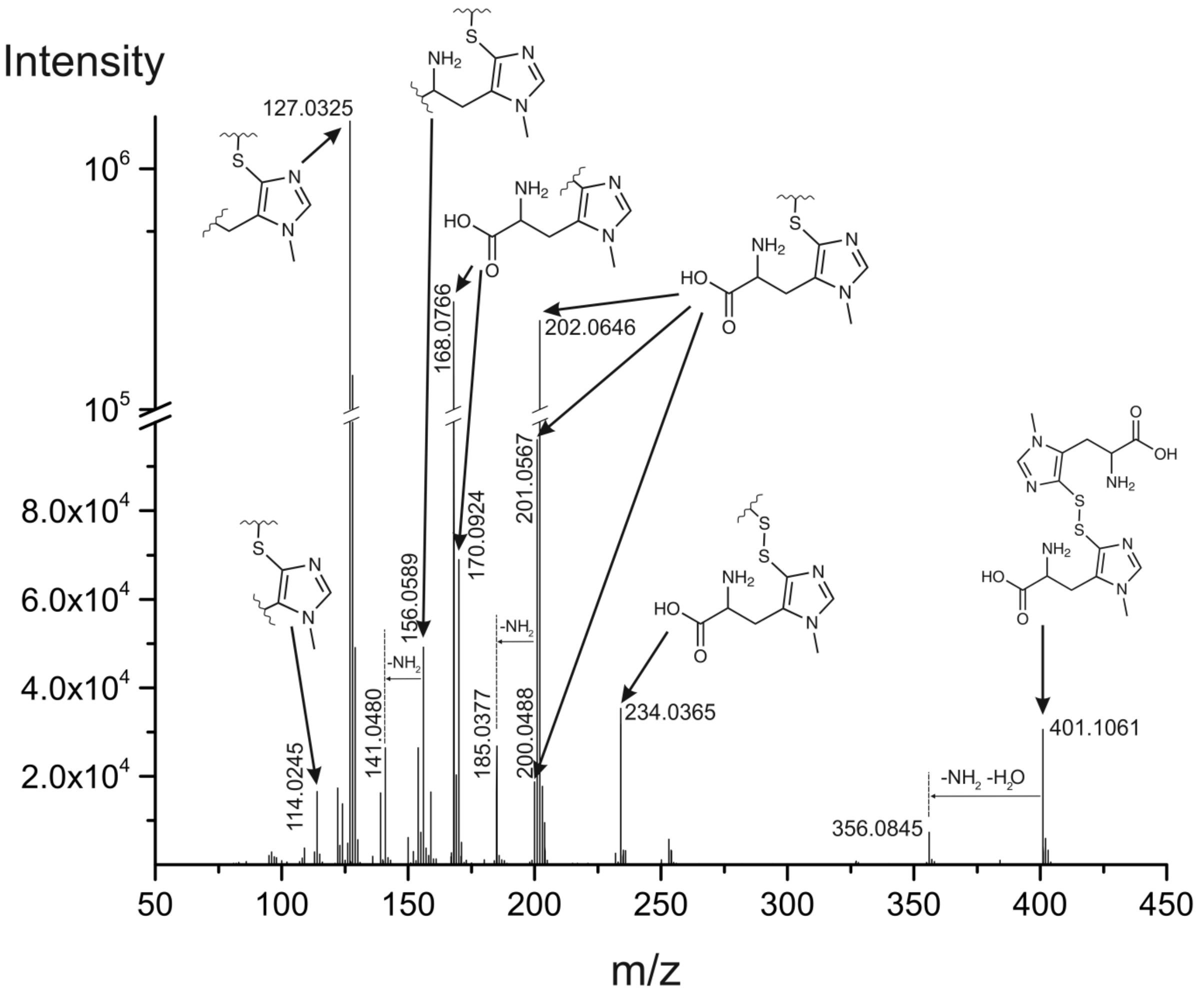

Figure S4. ESI-Q-TOF mass-spectrum of CID fragments (MS/MS) of isolated parent ion with m/z 401.1061 (OSSO). Collision energy 35eV, positive ion mode. Wavy lines indicate the positions of the bond cleavage.
